# The role of soluble mediators in the clinical course of EBV infection and B cell homeostasis after kidney transplantation

**DOI:** 10.1038/s41598-020-76607-z

**Published:** 2020-11-11

**Authors:** Sharon Bajda, Arturo Blazquez-Navarro, Björn Samans, Patrizia Wehler, Sviatlana Kaliszczyk, Leila Amini, Michael Schmueck-Henneresse, Oliver Witzke, Ulf Dittmer, Timm H. Westhoff, Richard Viebahn, Petra Reinke, Oliver Thomusch, Christian Hugo, Sven Olek, Toralf Roch, Nina Babel

**Affiliations:** 1 Berlin Institute of Health Center for Regenerative Therapies (BCRT): Berlin-Brandenburger Centrum für Regenerative Therapien, Charité-Universitätsmedizin Berlin, Berlin, Germany; 2 Systems Immunology Lab, Department of Biology, Humboldt-Universität zu Berlin, Berlin, Germany; 3 Medical Department I, Center for Translational Medicine, Marien Hospital Herne, University Hospital of the Ruhr-University Bochum, Herne, Germany; 4 Ivana Türbachova Laboratory for Epigenetics, Epiontis GmbH, Precision for Medicine Group, Berlin, Germany; 5 Chirurgical University Hospital, University Hospital Knappschaftskrankenhaus Bochum, University Hospital of the Ruhr-University Bochum, Bochum, Germany; 6 Berlin Center for Advanced Therapies (BeCAT), Charité-Universitätsmedizin Berlin, Berlin, Germany; 7 Department of Infectious Diseases, Institute for Virology, University Hospital Essen, University of Duisburg-Essen, Essen, Germany; 8 Department of General Surgery, University Hospital Freiburg, Freiburg, Germany; 9 Medical Clinic 3 – Nephrology Unit, University Hospital Carl Gustav Carus, Dresden, Germany

**Keywords:** Allotransplantation, Antimicrobial responses, Cytokines

## Abstract

Epstein-Barr virus (EBV) reactivation can lead to serious complications in kidney transplant patients, including post-transplant lymphoproliferative disorder (PTLD). Here, we have assessed the impact of EBV on B cell homeostasis at cellular and humoral level. In a multicenter study monitoring 540 kidney transplant patients during the first post-transplant year, EBV reactivation was detected in 109 patients. Thirteen soluble factors and B cell counts were analyzed in an EBV^+^ sub-cohort (N = 54) before, at peak and after EBV clearance and compared to a control group (N = 50). The B cell activating factor (BAFF) was significantly elevated among EBV^+^ patients. No additional soluble factors were associated with EBV. Importantly, in vitro experiments confirmed the proliferative effect of BAFF on EBV-infected B cells, simultaneously promoting EBV production. In contrast, elevated levels of BAFF in EBV^+^ patients did not lead to B cell expansion in vivo. Moreover, diminished positive inter-correlations of soluble factors and alterations of the bi-directional interplay between B cell and soluble factors were observed in EBV^+^ patients at peak and after clearance. Our data suggest that such alterations may counteract the proliferative effect of BAFF, preventing B cell expansion. The role of these alterations in lymphoma development should be analyzed in future studies.

## Introduction

Post-transplant lymphoproliferative disorders (PTLD) are a potentially fatal malignancy and one of the most severe complications after transplantation^1,2^. While it comprises a highly heterogeneous range of manifestations including asymptomatic, spontaneously regressing B cell expansion, most PTLD occur as lethal, fulminant lymphomas^1,2^. The majority of PTLD cases are driven by an infectious agent, the Epstein-Barr virus (EBV)^1,3^.

EBV is a nearly ubiquitous γ-1 herpesvirus, infecting approximately 90% of the healthy population^3–5^. EBV primary infection commonly occurs in early childhood and targets B cells, establishing latency in the host memory B cells^3,6–8^. The use of immunosuppression can disrupt the delicate balance between immune system and latent virus control, causing a 50-fold increase of EBV-infected B cells in peripheral blood^9,10^. As a consequence, EBV reactivation is a common occurrence after solid organ transplantation^11,12^.

In renal transplantation, about 20% of the patients develop EBV viremia during the first post-transplantation year. Seronegative patients and/or patients under immunosuppressive treatment with antithymocyte globuline are especially prone^11,12^. On the other hand, EBV-associated PTLD occurs in only 0.5–2.0% of kidney transplantation patients^1,2^. This discrepancy can be partially explained by the degree of severity of the EBV reactivation, with higher viral loads being associated with an increased risk of the disease^1,13,14^. However, the factors determining why certain EBV reactivations cause PTLD while others remain asymptomatic still remain elusive^1,15^.

In this study, we have assessed the impact of EBV reactivation on B cell homeostasis at the cellular and humoral level in a cohort of patients during the first year after renal transplantation. Thus, we investigated the effect of reactivation on the kinetics of three soluble factors associated with PTLD development: interleukin (IL)-6, IL-10 and the tumor necrosis factor (TNFα)^15–18^. Furthermore, additional soluble factors with a known role in B cell differentiation, proliferation, activation and class switch recombination or somatic hypermutation were analyzed, including IL-4, IL-5, IL-21, a proliferation-inducing ligand (APRIL) and the B cell activating factor (BAFF)^19–21^. The expression levels of these factors were correlated with the counts of circulating B cells, determined by epigenetic B cell counting, which provides robust cell quantification even in peripheral blood samples stored for a longer time period^22^.

Here, we provide evidence of profound changes in the interrelations between the humoral and the cellular levels among patients with EBV reactivation. We hypothesize that these changes could potentially explain the lack of B cell expansion and PTLD development.

## Material and methods

### Patient population

As published before, the viral monitoring was a sub-study on the cohort of the multicenter, open-label, randomized and controlled Harmony trial (NCT 00724022)^12,23^. The study was carried out in compliance with the Declaration of Helsinki and Good Clinical Practice. All participants provided written informed consent prior to inclusion into the study. The trial was approved by the Ethics Committee of Gustav Carus Technical University Dresden.

According to study design, induction therapy included either basiliximab or antithymocyte globuline (ATG). The maintenance immunosuppression was tacrolimus (Advagraf, Astellas) and mycophenolate mofetil (MMF)-based + /− corticosteroids^23^.

A total of 540 patients were monitored for EBV viral load, as well as cytomegalovirus and BK virus, along eight pre-defined visits during the first post-transplantation year; 3715 blood samples were analysed^12^. Monitoring was centrally performed^12^. The detection limit was 250 copies/mL^12^.

### Study design

**Figure 1:**
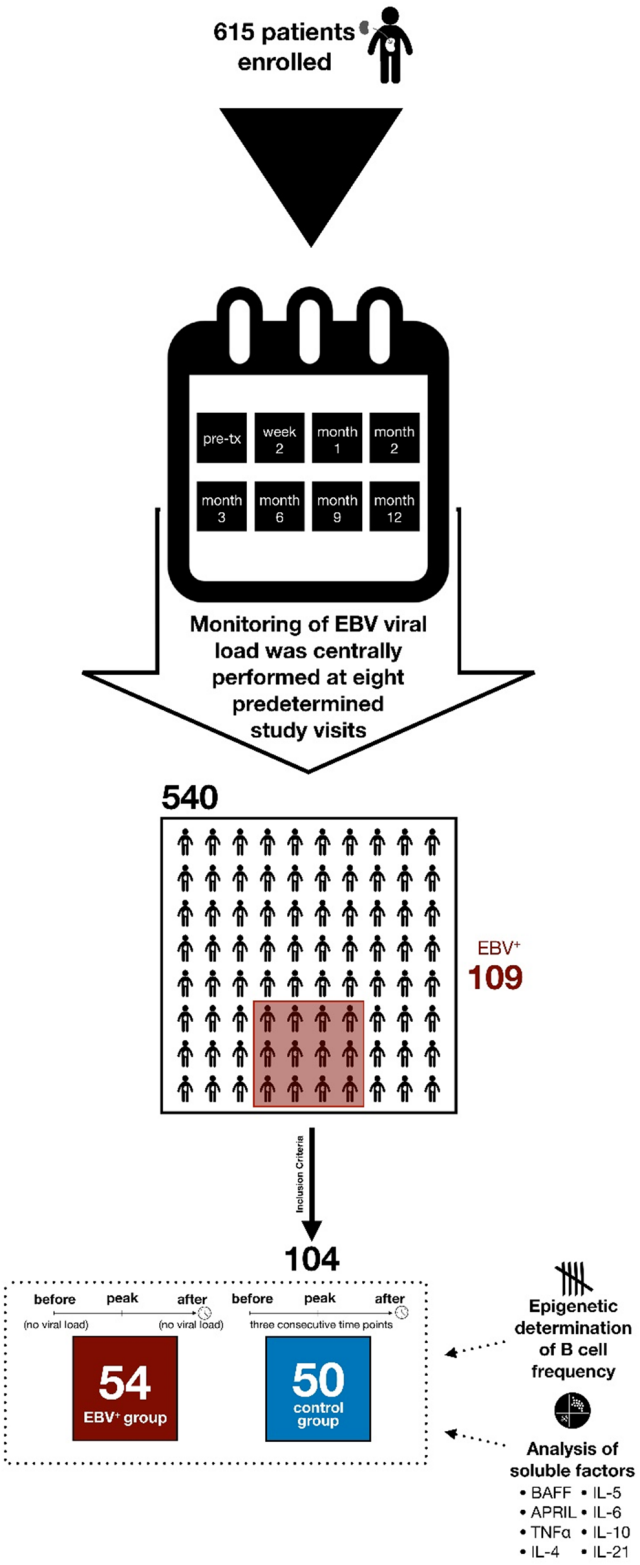
Trial profile. The diagram summarizes the viral monitoring performed in the Harmony cohort, the composition of the current cohort and the performed measurements.

The patient sub-cohorts were defined according to their peak of EBV viral load. All patients with at least one positive measurement for EBV viral load and available blood and serum samples at three time points (before viremia onset, at peak and after clearing) were recruited into the study and assigned into the EBV^+^ group. The control group was randomly selected among the patients with no detectable EBV viral load. The patients of both groups were matched according to demographic characteristics (sex, age of donor and recipient, body mass index and baseline blood cell count as well as C reactive protein concentration), therapy (therapy arm and anti-cytomegalovirus prophylaxis) and screening time points (see Table 1). Peripheral blood of EBV^+^ patients and the control group were analyzed at three time points for B cell frequency and concentration of the following soluble factors: IL-4, IL-5, IL-6, IL-10, IL-21, TNFα, APRIL and BAFF (see Fig. 1).

**Table 1:** Summary of the patient sub-cohorts characteristics.

Variable		Control group (N = 50)	EBV^+^ group (N = 54)	P value
Female gender		13 (26.0%)	15 (27.8%)	1.000
Age (years)		56 (44–62)	55 (49–63)	0.530
Donor age (years)		54 (43–61)	54 (48–62)	0.574
Body mass index (kg/m^2^)		25.2 (22.8–28.8)	24.8 (22.7–29.8)	0.925
Baseline white blood cell count (10^9^ cells/L)		7.4 (6.2–8.9)	6.7 (5.6–9.0)	0.265
Baseline C reactive protein (mg/L)		2.8 (0.5–5.5)	1.6 (0.5–3.0)	0.298
Negative EBV serostatus pre-transplant^a^		3 (10.3%)	1 (3.6%)	0.611
Therapy arm	Arm A (basiliximab + tacrolimus + MMF + steroids)	14 (28.0%)	15 (27.8%)	0.998
	Arm B (basiliximab + tacrolimus + MMF + rapid steroid withdrawal)	9 (18.0%)	10 (18.5%)	
	Arm C (rabbit ATG + tacrolimus + MMF + rapid steroid withdrawal)	27 (54.0%)	29 (53.7%)	
Anti-cytomegalovirus prophylaxis		27 (54.0%)	32 (59.3%)	0.732
Acute rejection incidence		7 (14.0%)	8 (14.8%)	1.000
Cytomegalovirus reactivation incidence		9 (18.0%)	13 (24.1%)	0.605
Glomerular filtration rate 2 weeks post-transplantation (mL/min/1.73 m^2^)		31.3 (17.8–43.7)	29.4 (15.9–47.9)	0.896
Glomerular filtration rate 1 year post-transplantation (mL/min/1.73 m^2^)		46.9 (35.6–64.5)	47.5 (33.3–58.9)	0.419
Screening time points (post-transplantation days)	Before	15 (1–56)	13 (1–54)	0.486
	Peak	31 (25–92)	30 (20–91)	0.623
	After	62 (55–247)	70 (58–250)	0.649

Details on the demographic and treatment characteristics employed for patient matching are given, as well as the transplantation outcomes. Additionally, details on the measurement schema of the present study are provided. Data are given in number (percentage) or median (IQR). P value is calculated employing Pearson’s chi-square test for binary variables and the Mann–Whitney test for continuous variables.

^a^EBV serostatus was available for 54.8% of cases.

The time points of sampling were defined according to the course of EBV infection: EBV^+^ patients were analyzed before reactivation (“before”), at peak (“peak”) and after clearance (“after”). Accordingly, the time points before and after reactivation had no detectable viral loads. For patients with multiple EBV^+^ samples, “peak” was defined as the sample with highest viral load; the “before” and “after” samples were selected considering all EBV^+^ samples, Therefore, “before” samples corresponded to samples before the first EBV positive sample and “after” samples were samples after the last EBV positive sample. For the control group, the time points were matched according to the time points of EBV^+^ patients. For reasons of comprehensibility, the terms for these three consecutive time points will be also “before”, “peak” and “after”.

### Screening of EBV viremia

As previously described by our group^12,24^ the EBV viral load was determined by quantitative polymerase chain reaction (qPCR) using the Prism 7500 Real Time Per System (ABI). PCR amplification were set up in a reaction volume of 25 µL using primer and probe at final concentrations of 900 nM and 5 µM. DNA was isolated from whole blood samples using the QIAamp DNA Mini Kit and according manufacturer's instructions (Qiagen Corp, Hilden, Germany).

### Epigenetic determination of B cell frequency

Due to the limited sample material availability, quantification of B cells could not be performed by conventional flow cytometry methods. Therefore, B cell frequency was determined based on the epigenetic quantification demonstrating a high correlation level with flow cytometry data as previously described by Baron et al*.*^22^ Briefly, frozen blood samples were thawed and lysed by addition of AL buffer and protease (QIAamp DNA MiniKit, Qiagen) at 56 °C for 1 h. After lysis, absolute ethanol was added to the lysate followed by washing and elution steps according to the manufacturer’s instructions. For bisulfite conversion and purification, genomic DNA was converted for 45 min at 80 °C in the presence of ammonium bisulfite and tetrahydrofuryl alcohol. Converted DNA was purified by magnetic beads (Mag-Bind Blood & Tissue DNA HDQ 96 Kit) and both customized and adapted buffers.

For epigenetic qPCR, following conditions were applied: Thermal cycling 1 × 95 °C for 10 min, followed by 50 cycles 95 °C for 15 s, and 61 °C for 1 min in 10 µl-reactions using the Roche LightCycler 480 Probes Master.

### Analysis of soluble factors

Analysis of soluble factors was performed as previously described by Stervbo et al.^25^ Briefly, soluble mediators including human IL-4, IL-5, IL-6, IL-10, IL-21, TNFα, BAFF, and APRIL were assessed using the LEGENDplex custom panel (BioLegend, San Diego, CA, USA). The samples were processed following the manufacturer’s instructions. In short, 1:2 diluted serum samples were mixed with assay beads in a V-bottom microtiter plate and incubated overnight. After washing and centrifugation, biotinylated detection antibodies were added following incubation for 1 h at 600 rpm on a plate shaker at room temperature. Streptavidin-PE detection antibodies were added and incubated for 30 min at 600 rpm at room temperature. After finial washing, samples were measured. Quantification of the Streptavidin-PE signal fluorescence intensity was done using a flow cytometer (CytoFlex S, Beckman Coulter, Germany). From the resulting fcs-files, the analyte concentration was extracted using LEGENDplex Data Analysis Software.

### Generation of lymphoblastic cell lines, quantification of B cells and EBV load under BAFF treatment

Lymphoblastic cell lines (LCL) have been generated from PBMC donated by kidney transplant patients as previously described^26^. In brief, PBMC were treated with B95-8 EBV containing viral supernatants in the presence of CpG OND2006 (2.5 µg/ml) and cyclosporine A (1 mg/ml). To study the effect of BAFF on the LCL, 5 × 10^5^ cells were cultured in a 96 well flat bottom plate and treated with BAFF (1 ng/ml, 10 ng/ml and 100 ng/ml) in presence or absence of mycophenolic acid (MPA) (2.5 µg/ml) and tacrolimus (Tac) (6 ng/ml). The drug concentrations in cell culture corresponded to the therapeutic trough level. Cell counts of live LCL were determined by flow cytometry (Cytoflex LX, Beckman Coulter). The viral load was determined in pools of the cell lysate and the cell culture supernatants using real time PCR as described in previous paragraph.

### Statistical analysis

Differences between groups for categorical variables were calculated using Pearson’s chi-square test with continuity correction. Differences in quantitative variables between groups are analysed using the two-tailed Mann–Whitney U test; the expansion of B cells was tested employing the two-tailed Wilcoxon signed-rank test with μ = 0. Correlation size and significance were calculated using Spearman’s correlation coefficient. The equality between two correlation matrices was tested employing the Jennrich test^27^. The effects of BAFF on in vitro viral replication and B cell expansion were evaluated univariately through the Wilcoxon signed-rank test and multivariately through linear regression, controlling for the effect of immunosuppression and patient-specific effects. Normalized LCL B cell count was calculated for each patient dividing the B cell counts for every condition by the B cell count for untreated (no BAFF and no immunosuppression) cells. Real time data on EBV load were calculated as the inverse of the real time PCR threshold, normalized using the untreated condition (No BAFF, no immunosuppression) as control. In all cases, P values below 0.050 were considered significant. P values were not corrected for multiple testing, as this study was of exploratory nature^28–30^.

Categorical variables are summarised as numbers and frequencies; quantitative variables are reported as median and interquartile range (IQR). Box plots depict the median, first and third quartile of a variable; the maximum length of the whiskers corresponds to 1.5 times the IQR.

## Results

### Characteristics of the patient cohort

We analyzed the EBV viral load in a total of 3715 blood samples collected from 540 patients during the first post-transplantation year^12^. As described before, EBV viral load was detected in 109 (20.2%) patients; 37 (6.9%) patients suffered from elevated viral load (> 2000 copies/mL) while high-level viremia (> 10,000 copies/mL) was observed in 11 patients (2.0%)^12^.

104 patients (54 EBV^+^ patients) of the original cohort were analyzed in-depth in this work. 18 patients (33.3%) had detectable viral load for more than one visit; only two patients (3.7%) had a prolonged viremia during more than two visits. There was only one case of severe EBV^+^ PTLD with a peak viral load of 12,271 copies/mL in the cohort^12^. Unfortunately, this PTLD patient withdrew his informed consent for the study participation and could not therefore be included into the EBV^+^ sub-cohort for the present work. Details on the demographic characteristics, employed treatment, transplantation outcomes and screening schema of the groups are summarized in Table 1. Importantly, there were no significant differences between the two sub-cohorts for any of these characteristics.

### BAFF was significantly associated with EBV reactivation

**Figure 2:**
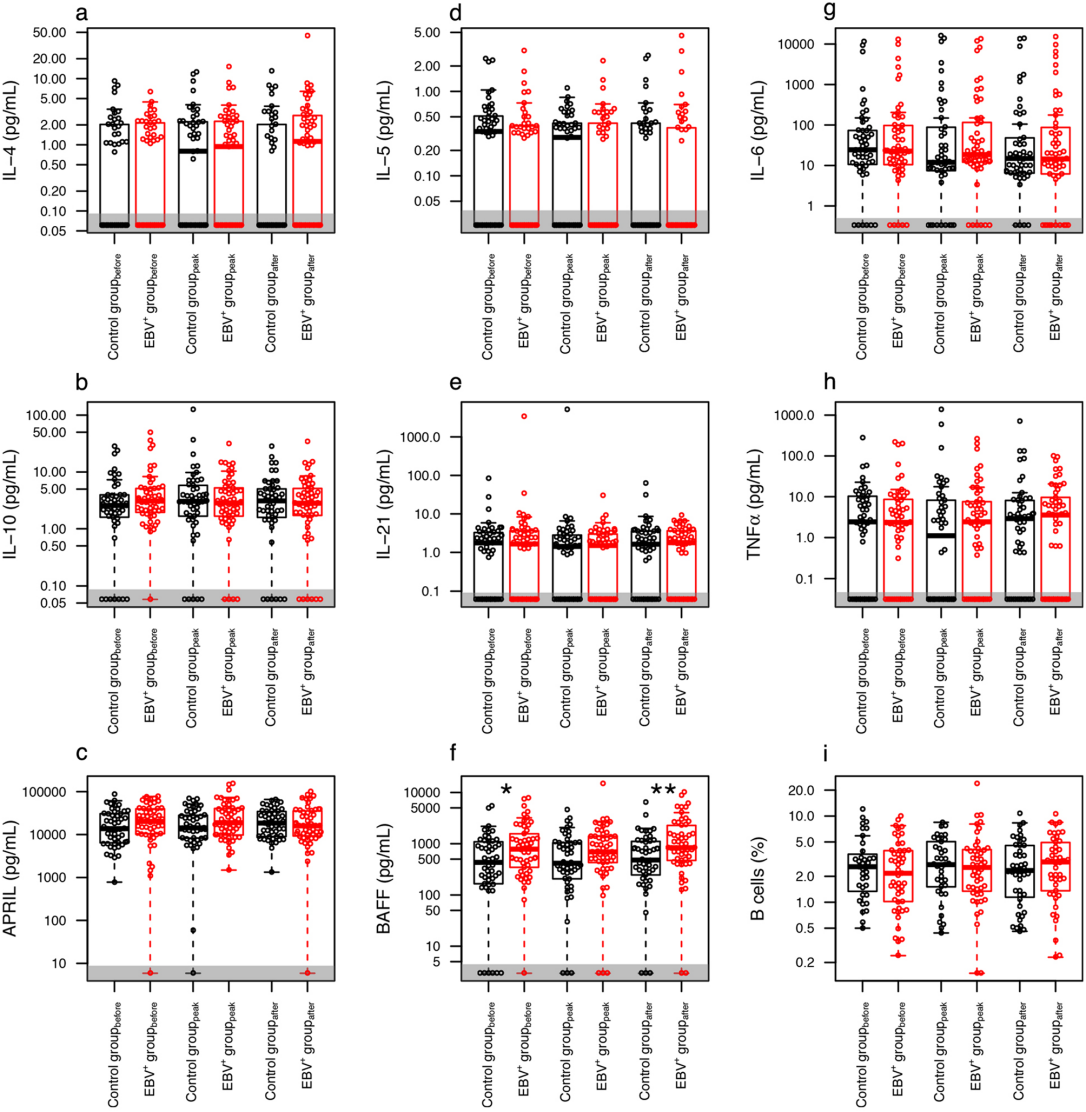
Concentration of the soluble mediators and B cell frequencies at the three time points. Patients in the control group are depicted in black, EBV^+^ patients in red. Significant differences (P < 0.050) between the sub-cohorts are indicated with one asterisk, highly significant differences (P < 0.010), with two asterisks. Note that the y-axis is in logarithmic scale. For the sake of convenience, measurements below detection limit are depicted with an arbitrarily defined low value within the area shaded in gray; for the calculation of significance these measurements were considered to have a value of 0.

In previous studies, we and others observed an association of IL-6, IL-10 and TNFα expression with EBV-associated PTLD^15–18^. Therefore, we analyzed whether these factors, as well as other B cell homeostasis associated soluble factors, can predict or contribute to the EBV reactivation. Additionally, we analyzed whether EBV reactivation itself can lead to changes in the soluble factor expression pattern. In total, 312 samples from 104 patients were measured for B cell frequency and IL-4, IL-5, IL-6, IL-10, IL-21, TNFα, APRIL and BAFF concentration (Fig. 2).

**Figure 3:**
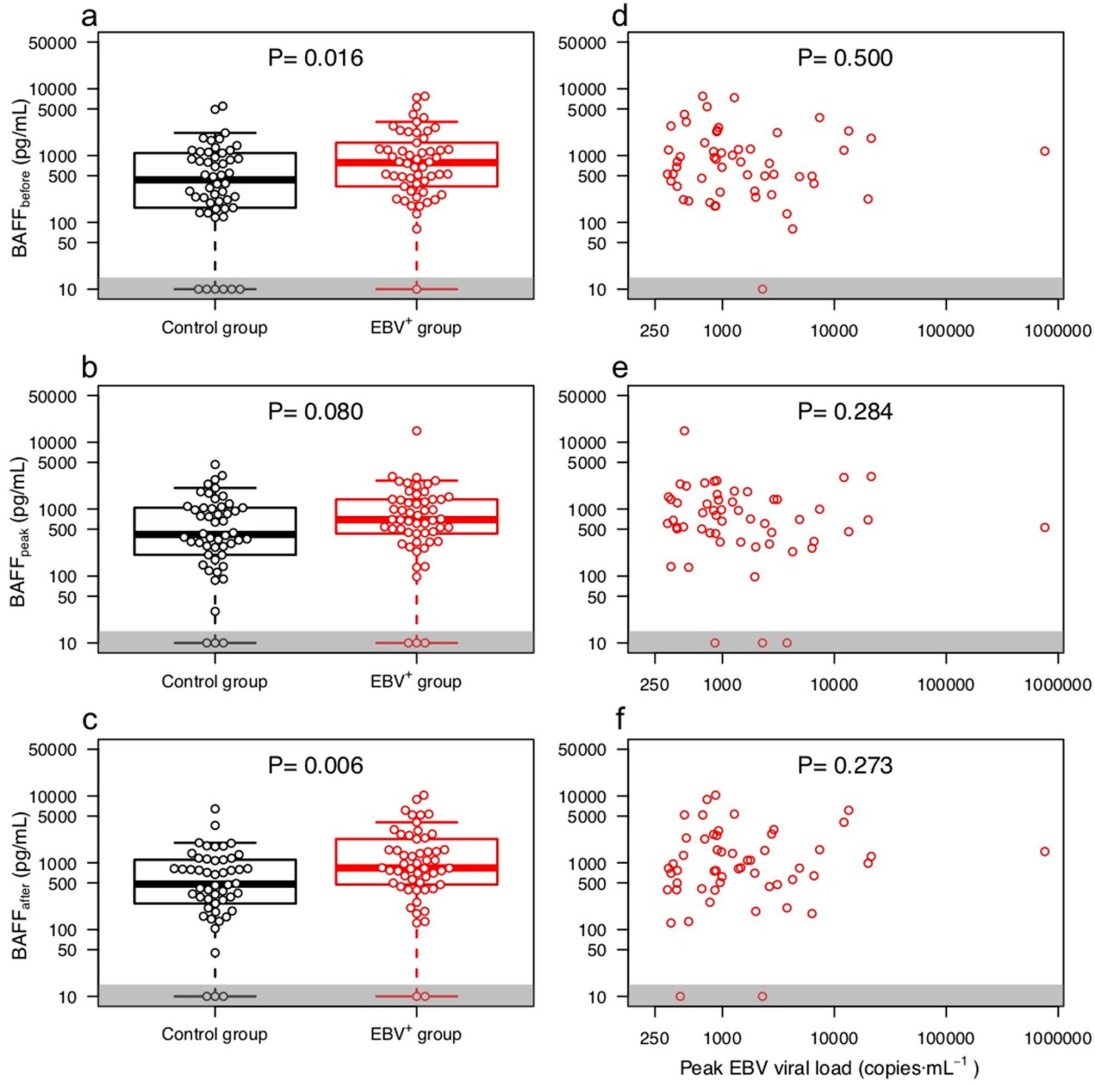
BAFF concentration in serum as a function of EBV reactivation. The left column shows the measured BAFF concentration for both sub-cohorts at the time points before **(a)**, peak **(b)** and after **(c)**; the right column depicts the measured BAFF concentrations in the EBV^+^ group at time points before **(d)**, peak **(e)** and after **(f)** as a function of EBV viral load at peak. The results on **(a–c)** are also contained in Fig. 1f and are shown here enlarged for a better data view. Samples with a BAFF concentration below detection limit are depicted in the gray area; for the calculation of significance these concentrations were considered to have a value of 0. Note that both axes are in logarithmic scale.

We observed a significant association between EBV positivity and increased levels of BAFF in serum. As shown in Fig. 3a–c, a significant association of EBV reactivation was found both before [784 (355–1482) vs. 433 (173–1053) pg/ml; P = 0.016] and after reactivation [836 (478–2094) vs. 480 (253–1100) pg/ml; P = 0.006], while at peak only a borderline significant difference was observed [694 (432–1393) vs. 416 (222–1045) pg/ml; P = 0.080]. Remarkably, we did not find any correlation between peak EBV load and BAFF levels (Fig. 3d–f).

None of the other measured soluble factors demonstrated a significant association with EBV reactivation, nor a correlation with peak viral load (Fig. 2).

### In vitro BAFF treatment of EBV infected B cells facilitates their expansion and viral replication

To better understand the association of EBV with increased BAFF concentrations, we analyzed the in vitro effects of BAFF on lymphoblastoid cell lines (LCL) generated from kidney transplant patients by infecting their B cells with EBV. Upon generation of stable LCL from four patients, the cell lines were cultured with increasing concentrations of BAFF in the presence or absence of the immunosuppressive drugs MPA and tacrolimus.

**Figure 4:**
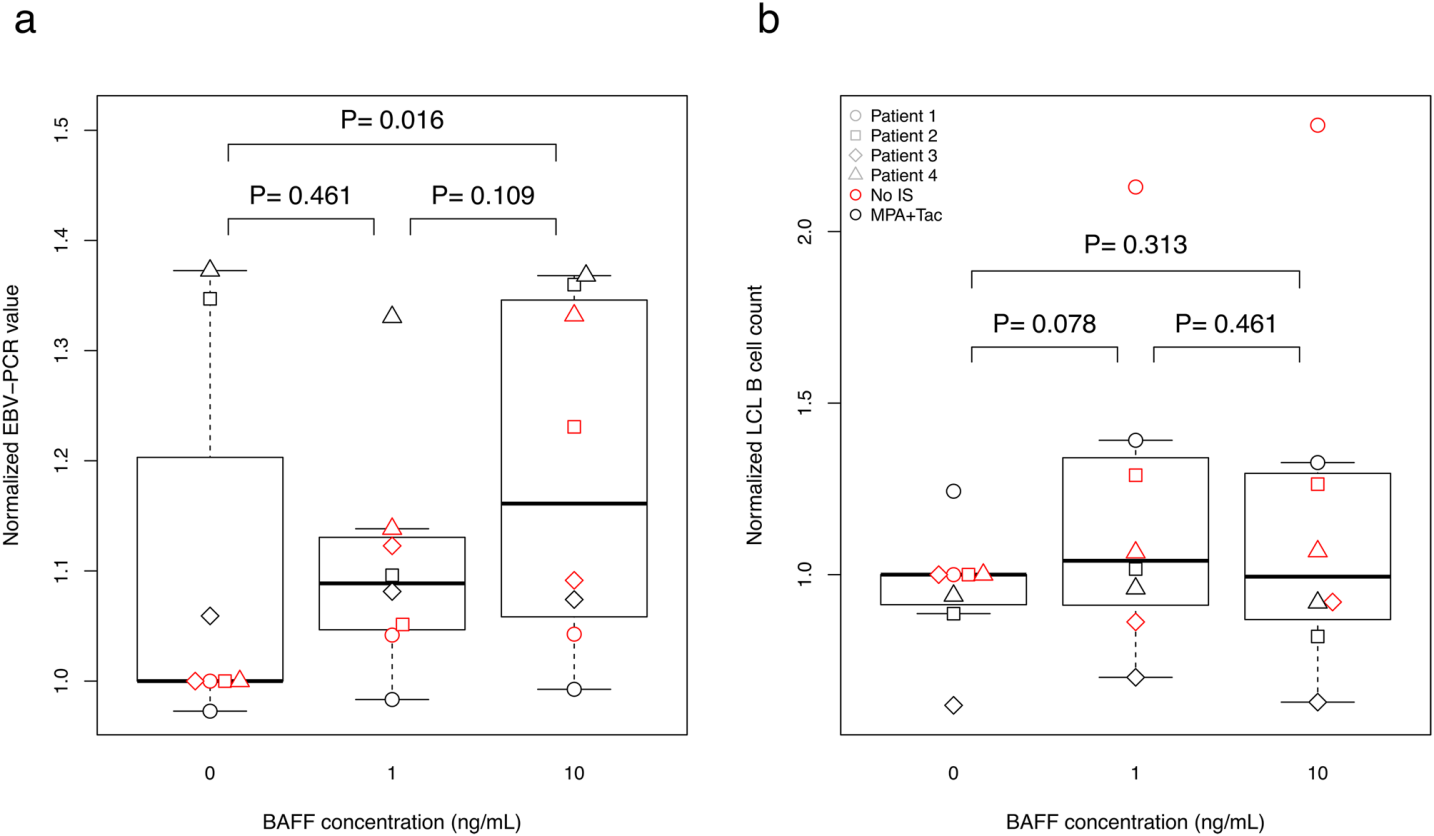
BAFF treatment of EBV-infected LCL leads to increased EBV replication and cell expansion. LCL B cells from four different renal transplant patients were cultured with increasing BAFF concentrations (x-axis), with MPA + Tac (black symbols), or without MPA + Tac (red symbols). EBV load under BAFF treatment was assessed by normalization of EBV-PCR data obtained after BAFF treatment to untreated samples **(a)**. The normalized LCL B cell count **(b)** represents the ratio of the B cell count by the untreated (BAFF = 0 and No IS) LCL B cells.

To analyze the impact of BAFF on B-cell proliferation and EBV replication, flow cytometry analysis and quantitative EBV-specific real-time PCR were performed, respectively. Our data demonstrate higher EBV load in LCL treated by BAFF compared to untreated controls (Fig. 4a). The observed difference was significant (0.13 ± 0.06, P = 0.041; see Table S1), as tested employing multi-parameter regression analysis. Likewise, although not significant in univariate comparison (Fig. 4b), BAFF treatment was significantly associated with increased numbers of LCL, compared to untreated cells as demonstrated by multi-parameter regression controlling for confounders (0.36 ± 0.14, P = 0.017; Table S1). However a dose dependent effect of BAFF could not be observed.

### EBV reactivation and BAFF expression did not lead to B cell expansion

**Figure 5:**
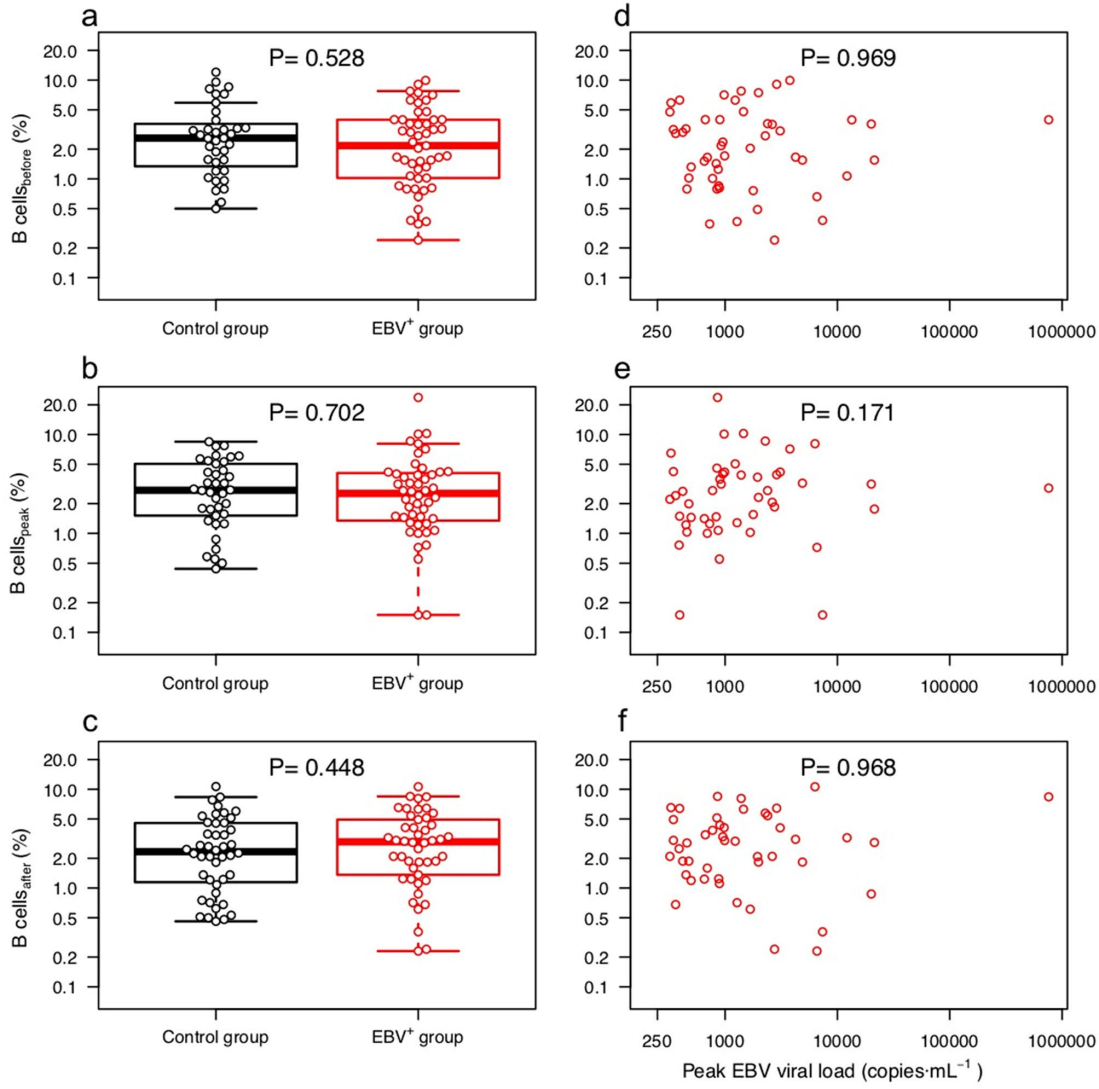
B cell frequency in blood as a function of EBV reactivation. The left column shows the measured B cell frequencies for both sub-cohorts at the time points before **(a)**, peak **(b)** and after **(c)**; the right column depicts the measured B cell frequencies in the EBV^+^ group at time points before **(d)**, peak **(e)** and after **(f)** as a function of EBV viral load at peak. The results on a-c are also contained in Fig. 1i and are shown here enlarged to allow for a better view of the data. Note that both axes are in logarithmic scale.

As BAFF was shown to be associated with increased viral replication and B cell proliferation in vitro, we wondered whether we observed a similar effect of EBV reactivation on the levels of peripheral B cells among EBV^+^ patients. The peripheral B cell was quantified by epigenetic cell counting, which shows a high correlation with frequently used flow cytometry determination of ρ = 0.96–0.98^22^. Surprisingly, no significant difference in B cell frequency was observed between the patients with EBV reactivation and the control group, at any of the three time points (see Fig. 5a–c). Likewise, no correlation between kinetics of EBV loads and B cell count kinetics was found within the EBV^+^ group (Fig. 5d–f). There was no significant B cell expansion among EBV^+^ patients neither at peak (P = 0.739) nor after reactivation (P = 0.543), compared with the time point before reactivation. Furthermore, no significant difference in the B cell kinetics was observed between the EBV^+^ patients and the control group (see Figure S1).

### EBV reactivation was significantly associated with an altered state of the immune system

**Figure 6:**
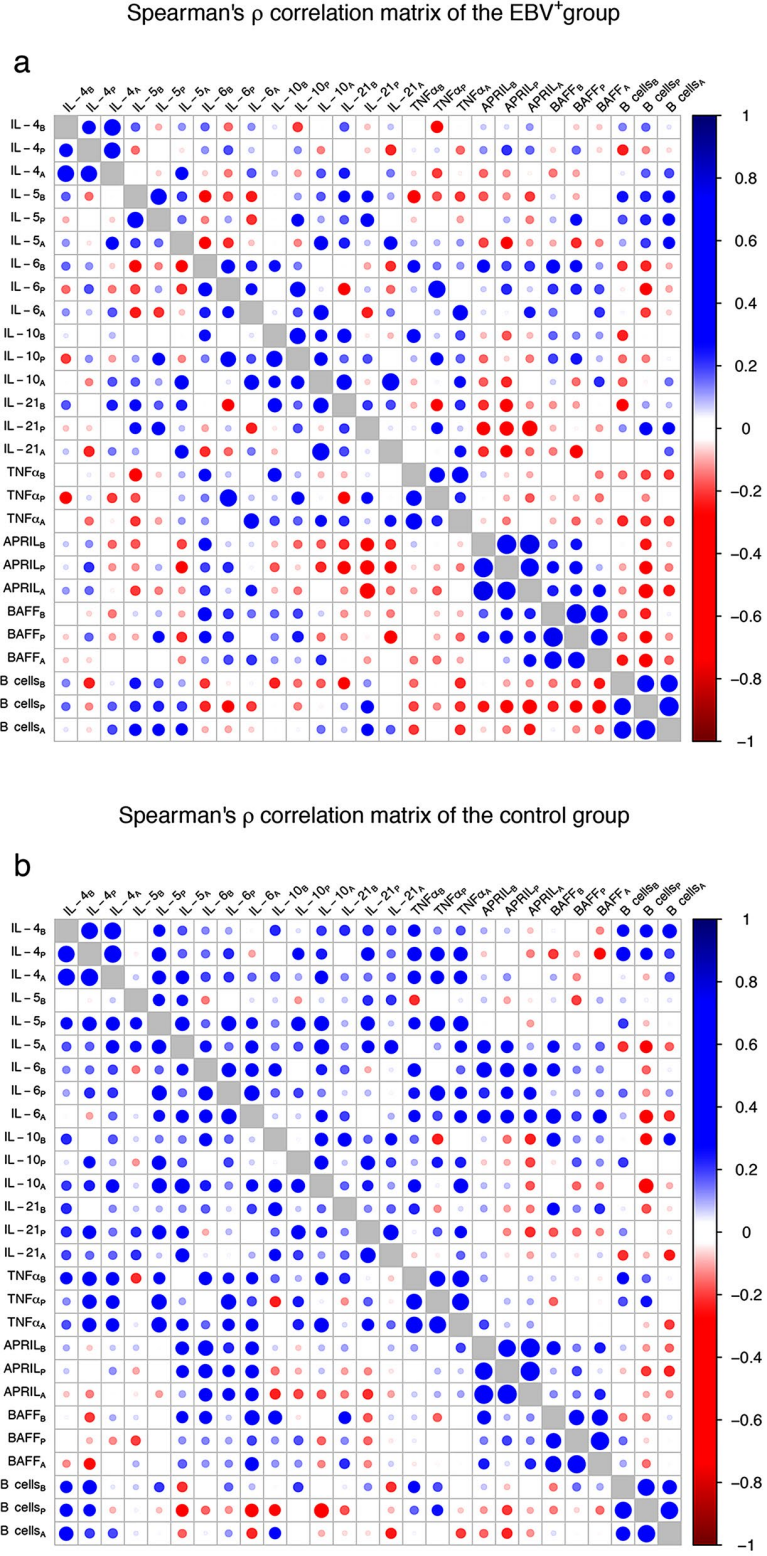
Correlation matrices of soluble factors and B cell for the patient sub-cohorts. **(a)** Represents the correlation matrix of the EBV^+^ group, whereas **(b)** depicts the correlation matrix of the control group. Correlation strength is represented by both circle size and color intensity, where (as shown in the legend) blue tones denote positive correlations and red tones, negative correlations. Subscripts in variable labels denote the time point of measurement (B: before, P: peak, A: after).

**Figure 7:**
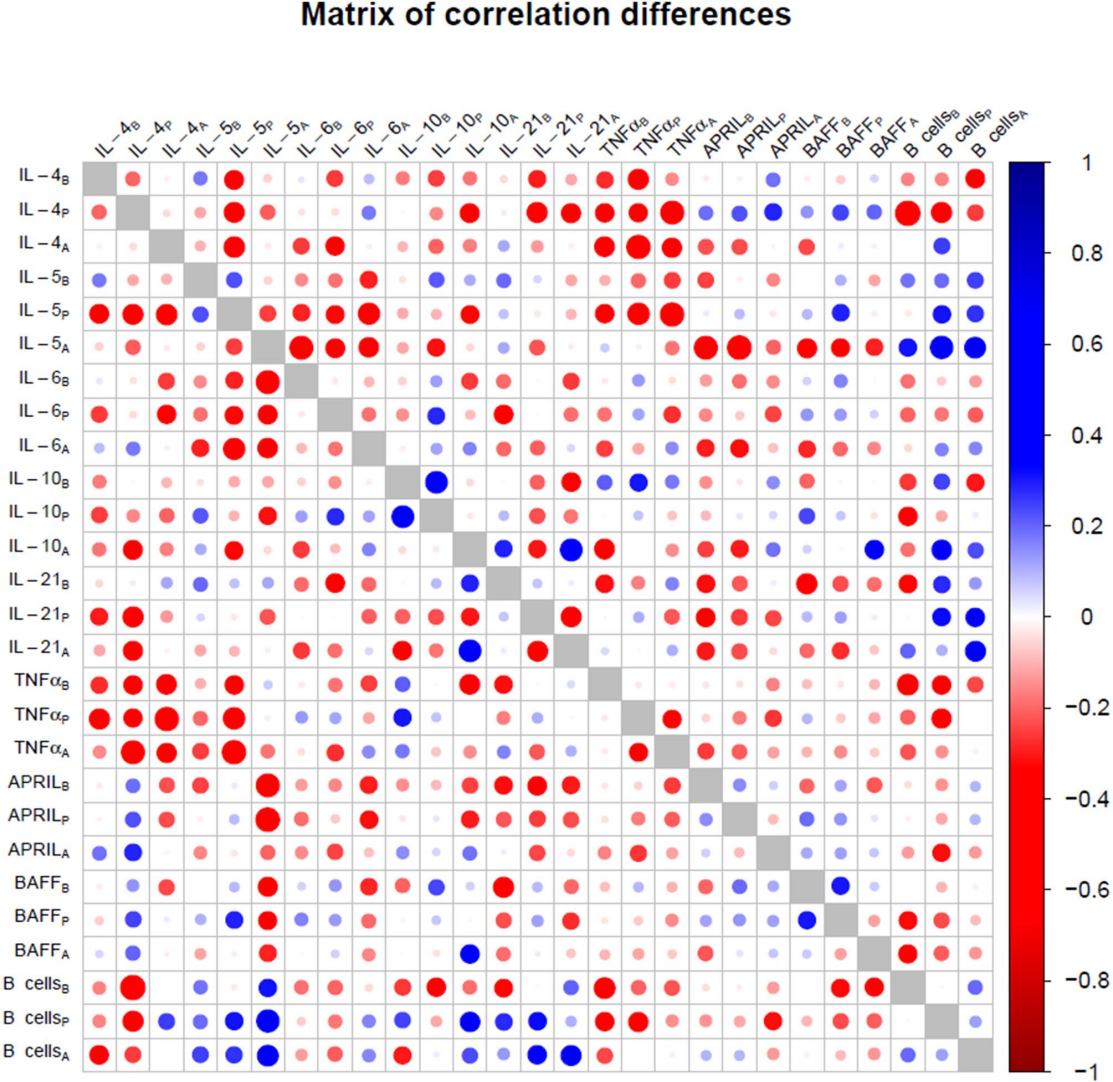
Difference between the correlation matrices of the EBV^+^ group and the control group. The absolute value difference between the correlations is represented by circle size. Blue tones denote that the EBV^+^ group had an increased correlation compared to the control group and vice versa for the red tones. Subscripts in variable labels denote the time point of measurement (B: before, P: peak, A: after).

Since no B cell expansion was observed despite increased BAFF level in EBV^+^ patients, we hypothesized that other soluble factors or soluble factor patterns might counteract the proliferative effect of BAFF. Therefore, we analyzed the effects of EBV reactivation in the inter-correlations of the measured factors. Figure 6a,b shows the correlation matrix for the two sub-cohorts, while Fig. 7 depicts the difference between the correlation matrices. Supplementary Figure S2 shows only the significant correlations (P < 0.050) for the two sub cohorts.

Patients in the EBV^+^ group showed remarkable differences in their correlations matrix compared with the control group. The difference between the two correlation matrices was highly significant (P < 0.001). In detail, EBV reactivation was associated with a general weakening of the correlations between soluble factors: While the measured interleukins and TNFα were positively correlated with each other at almost all time points (90.8%) in the control group, the ratio of positive correlations in the EBV^+^ group was substantially lower (66.0%).

These differences also affected the interplay between soluble factors and B cells. Thus, IL-5 and IL-21 were positively correlated with B cell in the EBV^+^ group, whereas a mixture of positive and negative correlations were observed in the control group. Interestingly, in the case of BAFF the correlation with B cell frequency was negative among EBV^+^ patients, but this was not the case for the control group. As a result, as seen in Fig. 7, the correlation coefficients for the EBV^+^ group were lower than for the control.

To determine whether the altered state of immune system could possibly be a consequence rather than a cause of EBV reactivation, we further analyzed the time dependence of this altered state (see Figure S3). Remarkably, no significant difference could be observed between the sub-cohorts before EBV reactivation (P = 0.257). A significant difference was observed already at peak (P = 0.010); this difference was even larger after reactivation (P < 0.001).

## Discussion

In this study, we have analyzed the tri-directional interplay between EBV reactivation, B cells and B cell-associated soluble serum mediators. Our main findings include:

- Patients with EBV reactivation demonstrated significantly increased concentrations of BAFF in serum, both before viremia onset and after its clearing. No significant association was found between severity of EBV reactivation and BAFF levels.
- In accordance with these findings, BAFF treatment was associated with increased EBV replication and expansion of EBV infected B cells in vitro.
- In spite of the increased serum BAFF levels found in EBV^+^ group, EBV reactivation was neither associated with increased B cell frequency nor with B cell expansion.
- EBV reactivation was associated with a significantly altered state of the immune system as assessed by correlation matrix analysis. This alteration of the immune system was detected at reactivation peak and after clearing.

By demonstrating increased level of BAFF in EBV^+^ group, we showed for the first time a possible effect of EBV reactivation on B cell homeostasis. Our analyses demonstrate furthermore that EBV reactivation might have a profound effect on immune response, altering the interplay between soluble factors and cellular immunity. The significance of the alteration increased even more after clearing of EBV viral load. Interestingly, even though increased BAFF concentrations have been shown to be associated with EBV reactivation both in vitro and in vivo, no distinctive correlation profile was observed before viremia onset.

What is the nature of this altered state of the immune system? The univariate testing of single factors found only a significant association of EBV^+^ patients with higher levels of BAFF. BAFF is known as a key regulator of B cell homeostasis^31–33^, and its effects on expansion of EBV-infected B cell could be confirmed in our in vitro experiment. Importantly, no upregulation of the markers for PTLD development including IL-6, IL-10 and TNFα was observed^15–18^. Furthermore, the examination of the correlation matrices and the matrix of correlation differences (Figs. 5 and 6) reveals two general phenomena: A weakening of the positive inter-correlations of most measured factors (especially IL-4, IL-5, IL-6, IL-10, IL-21 and TNFα) and alterations of the bi-directional interplay between B cell and soluble factors. These latter alterations affect, among others, the correlation of BAFF with B cell frequencies. This is remarkable, as BAFF has been shown to be a lymphoma marker in the immunosuppressive context such as AIDS^34^. The differences between our study and the study on AIDS patients could be explained by the impact of EBV infection, as EBV infection was not considered in the AIDS study. In contrast, we demonstrated that EBV reactivation was associated with a strong reduction of the BAFF-B cell correlation in our study, which may explain the lack of B cell expansion.

Based on these observations, we speculate that the observed alterations in the B cell-associated soluble factor network would comprise not only an EBV-induced disruption of the immune system, but also the response of the immune system against these very same disruptions. This hypothesis would have far-reaching implications. Accordingly, the alterations in B-cell soluble factor pattern could therefore be viewed as part of an anti-PTLD signature. While the nature and mechanism of this immune alteration signature are highly speculative, the elevated BAFF concentrations before and during EBV reactivation found in our study could perhaps be a marker for this anti-PTLD state. In line with this, BAFF was not associated with PTLD in a recent study in liver transplantation patients^35^. Moreover, BAFF has been even shown to play an important role in antiviral immune response^36^. Further studies are needed to assess the nature and persistence of the EBV-associated alterations in the B cell homeostasis, as well as their hypothetical relationship with development of PTLD.

Besides the postulated role in antiviral defense, BAFF upregulation might be employed as a predictive marker of EBV reactivation. Thus, our data demonstrate an elevated level of BAFF prior EBV reactivation and an association of BAFF with increased in vitro viral replication. These results are in line with previous reports on the upregulation of BAFF in vitro and in vivo in EBV-infected B cells^37,38^.

Remarkably, our results demonstrate that the univariate analysis of the measured factors cannot capture the complexity of the changes in the B cell homeostasis associated with EBV reactivation. The consequences of reactivation could only be detected through a systems approach that takes into account all the interrelations between the measured factors. A reason for this is that immune soluble factors exhibit mutual synergies and act in concert, and analysis techniques have to take into account these synergies^39–41^. Correlation analysis is a powerful tool to identify these synergies and therefore changes in complex signaling systems, identifying altered states that transcend the mere changes in concentration of single factors and treating the network of correlations as a whole^40,42,43^. In the field of immunological research, correlation analysis has been recently employed to elucidate changes in the cytokine signaling associated with immunomodulatory drugs, anti-bacterial immune response and glioma development, among others^40,42,43^.

This study has some limitations: Firstly, even though there is evidence that suggests a role of prolonged EBV viremia in the development of PTLD, we did not analyze the influence of viremia duration separately. This was due to the low incidence of prolonged EBV viremia, as only two patients had detectable viral load for more than two visits. Secondly, only three time points were analyzed, so that we do not have any data on the persistence of the observed alterations of the immune system among EBV^+^ patients. Furthermore, since the follow-up of the Harmony trial was of 1 year, we do not have information on whether any of the patients of our study developed PTLD after 1 year post-transplantation, as it generally the case in adult renal transplantation^2,44,45^. Unfortunately, the only EBV^+^ PTLD case in our study cohort could not be analyzed in depth, due to the patient’s decision to withdraw informed consent. Therefore, no samples were available. Finally, with only 13 measured factors, we might be missing important aspects of the immune response in our analysis: among others, the role of T cells in controlling EBV^+^ reactivation and expansion of infected B cells, which could be a potential explanation for the observed altered state of the immune system and hypothetical anti-PTLD signature^1^.

In conclusion, our work offers insights in the EBV-associated changes of the B cell homeostasis, both from a humoral and cellular point of view. These alterations were especially striking after viral clearing and comprised a reduction of the B cell proliferating effects of BAFF. As reactivation was simultaneously associated with increased BAFF levels, it can be speculated that the observed alterations correspond to an anti-PTLD signature, which could explain the lack of BAFF-associated B cell proliferation and consequently PTLD development in our study. Additional studies, including a larger number of factors and measurements, are required to further elucidate the nature and consequences of the EBV-associated alterations of the immune system in renal transplantation patients.

## Supplementary information

Supplementary Information.

## Data Availability

The data used to support the findings of this study are available from the corresponding authors upon request.
