# Supplementary material for: The role of soluble mediators in the clinical course of EBV infection and B cell homeostasis after kidney transplantation
Source: Sci Rep. 2020 Nov 11;10:19594. doi: 10.1038/s41598-020-76607-z (PMC7658229; doi:10.1038/s41598-020-76607-z)
Supplement: Supplementary file 1 — Supplementary Information. [file 41598_2020_76607_MOESM1_ESM.pdf]

The role of soluble mediators in the clinical course of EBV infection and B cell homeostasis after kidney transplantation

Supplementary Material

Sharon Bajda[1], Arturo Blazquez-Navarro[1,2,3], Björn Samans[4], Patrizia Wehler[1,3], Sviatlana Kaliszczyk[1,3], Leila Amini[1],[6], Michael Schmueck-Henneresse[1,6], Timm H. Westhoff[3], Richard Viebahn[5], Petra Reinke[1,6], Oliver Witzke[7], Ulf Dittmer[7], Oliver Thomusch[8], Christian Hugo[9], Sven Olek[4], Toralf Roch[1,3], Nina Babel[1,3*]

[1]Berlin Institute of Health Center for Regenerative Therapies (BCRT): Berlin-Brandenburger Centrum für Regenerative Therapien, Charité-Universitätsmedizin Berlin, Berlin, Germany.

[2]Systems Immunology Lab, Department of Biology, Humboldt-Universität zu Berlin, Berlin, Germany.

[3]Center for Translational Medicine, Medical Department I, Marien Hospital Herne, University Hospital of the Ruhr-University Bochum, Herne, Germany

[4]Ivana Türbachova Laboratory for Epigenetics, Epiontis GmbH, Precision for Medicine Group, Berlin, Germany

[5]Chirurgical University Hospital, University Hospital Knappschaftskrankenhaus Bochum, University Hospital of the Ruhr-University Bochum, Bochum, Germany

[6]Berlin Center for Advanced Therapies (BeCAT), Charité-Universitätsmedizin Berlin, Berlin, Germany.

[7]Universitätsklinikum Essen, Department of Infectious Diseases and Institute for Virology, Essen, Germany.

[8]Department of General Surgery, University Hospital Freiburg, Freiburg, Germany.

[9]University Hospital Carl Gustav Carus, Medical Clinic 3 – Nephrology Unit, Dresden, Germany.

***Corresponding author: nina.babel@charite.de**

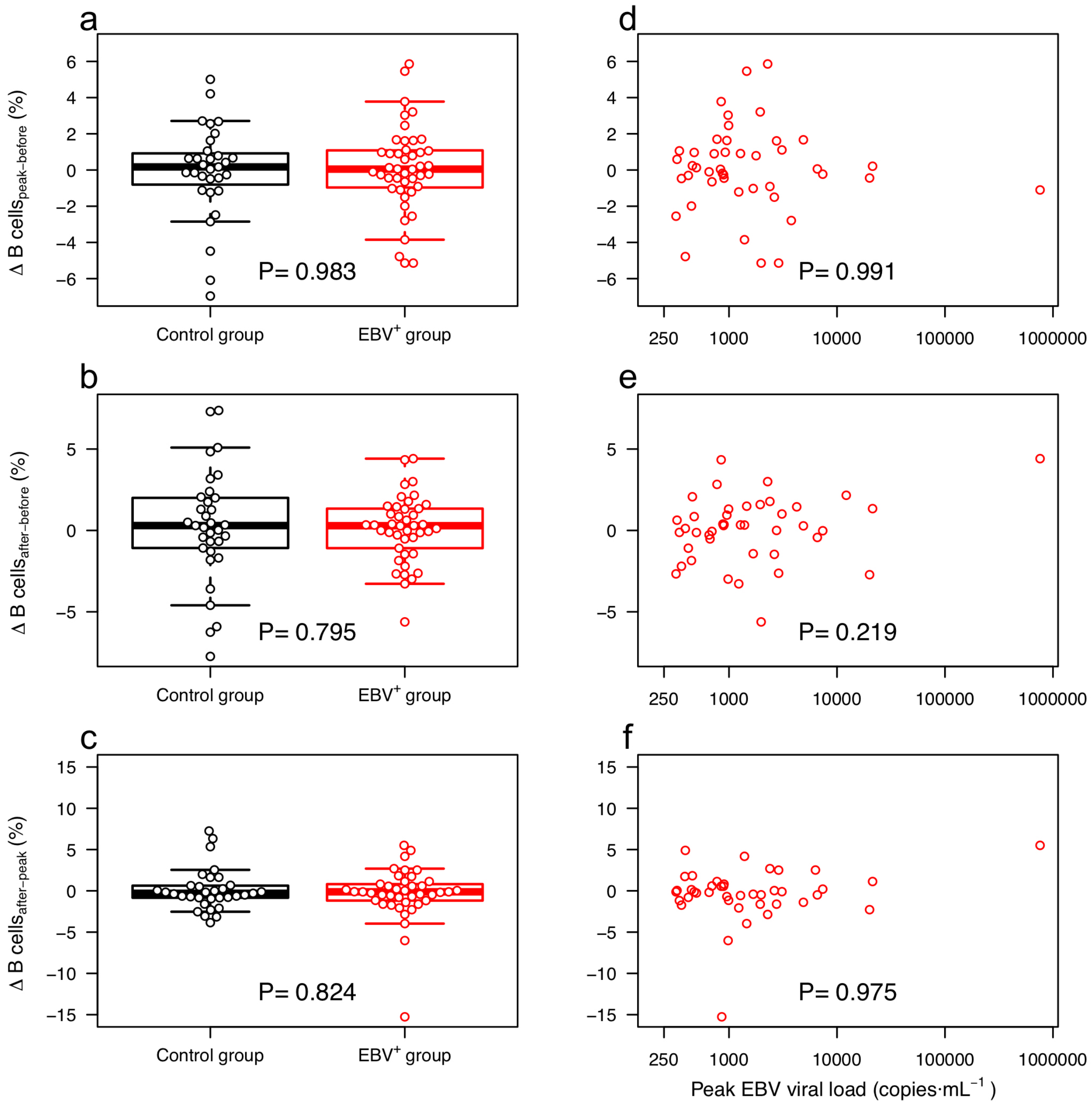

Figure S1. B cell kinetics as a function of EBV reactivation. The left column shows the increase in B cell frequency for each patient for the combination of time points peak-before (a), after-before (b) and after-peak (c); the right column depicts likewise the increase of B cell frequency in the EBV$^+$ group (d-f) as a function of EBV viral load at peak.

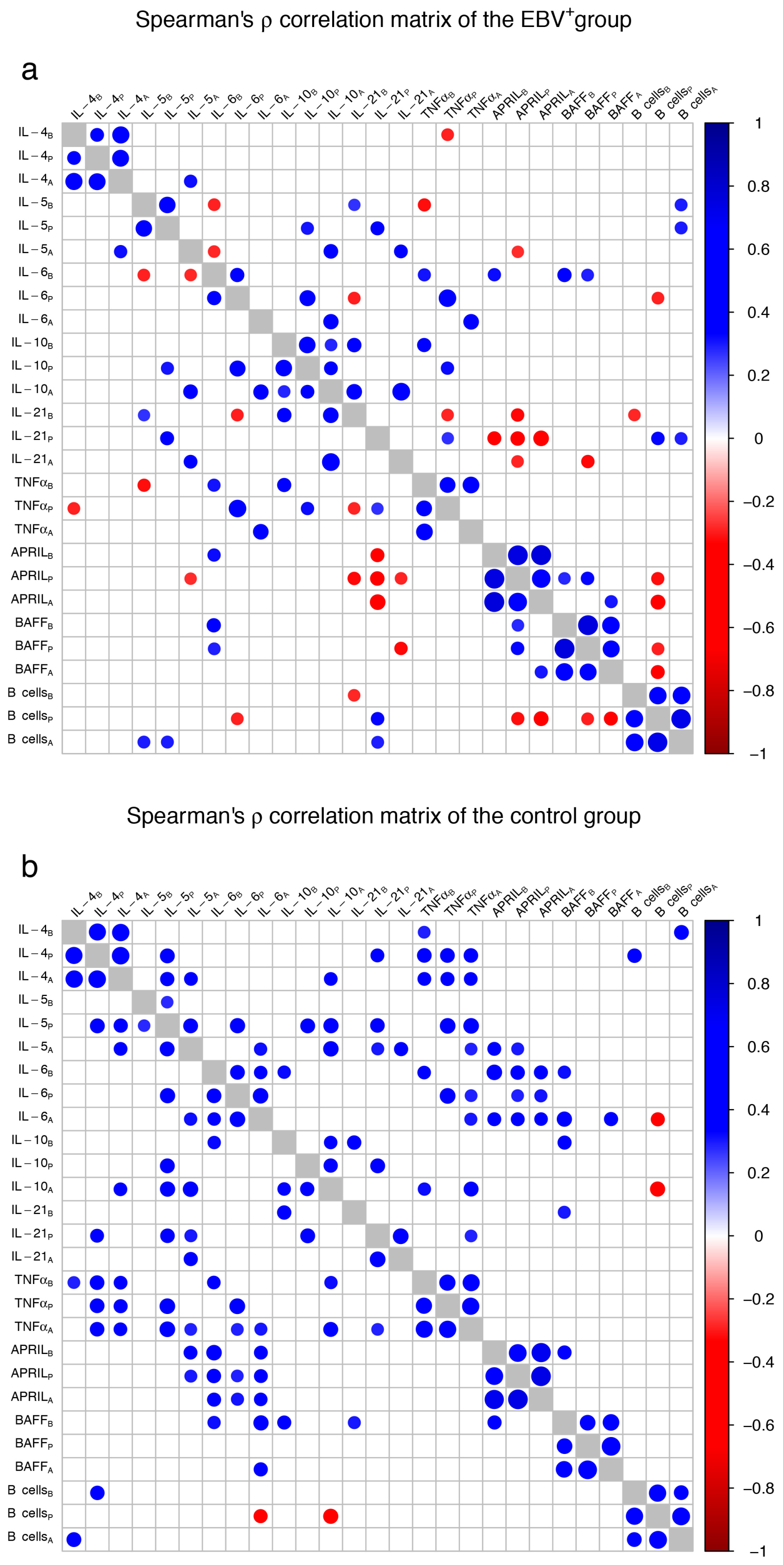

Figure S2. Significant correlation matrices of soluble factors and B cell for the patient sub-cohorts ($P<0.05$). S2a represents the correlation matrix of the EBV^{+} group, whereas S2b depicts the correlation matrix of the control group. Correlation strength is represented by both circle size and color intensity, where (as shown in the legend) blue tones denote positive correlations and red tones, negative correlations. Subscripts in variable labels denote the time point of measurement (B: before, P: peak, A: after).

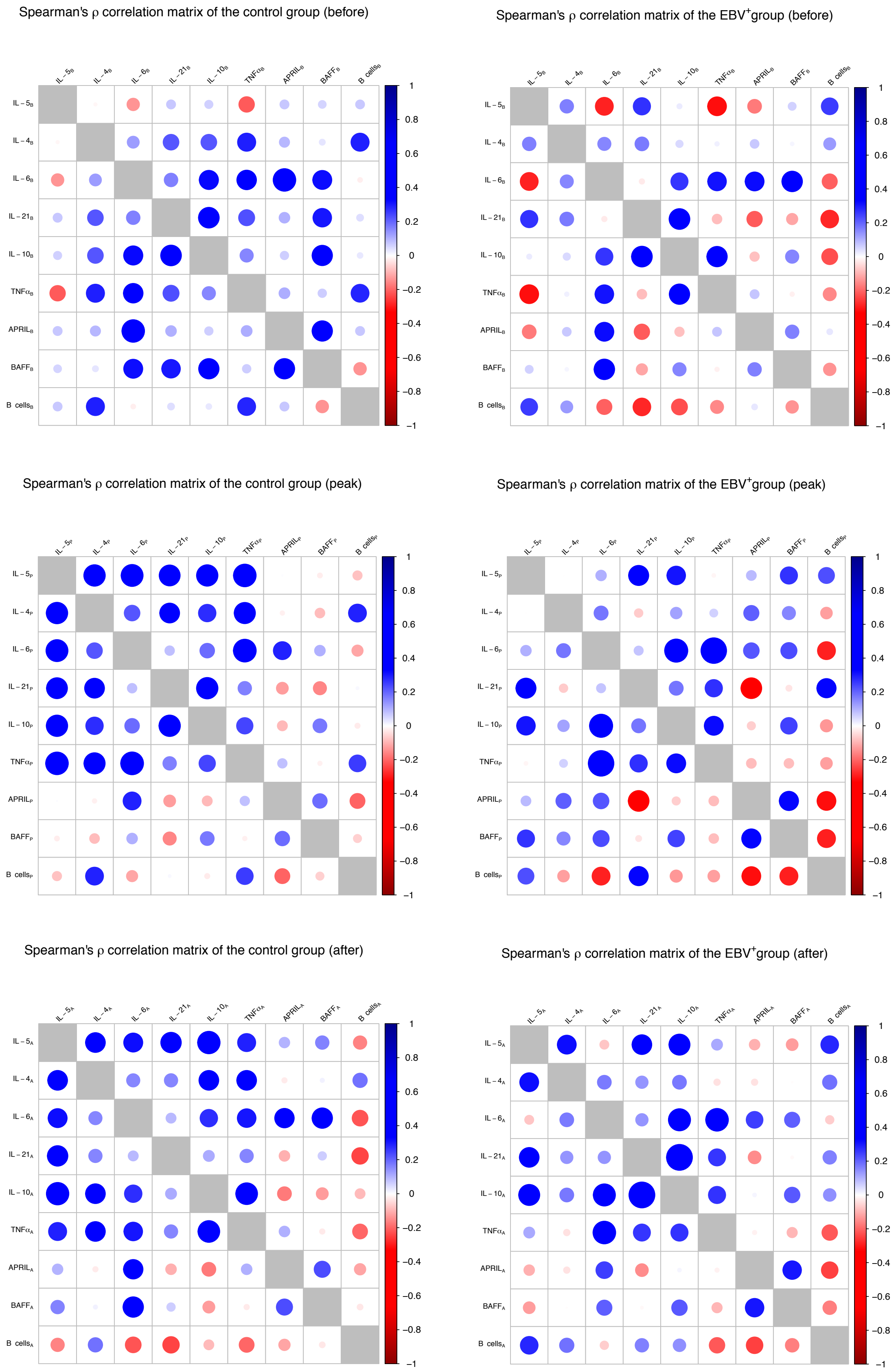

Figure S3. Correlation matrices of soluble factors and B cell for the patient sub cohorts stratified by measurement time point. Correlation strength is represented by both circle size and color intensity, where (as shown in the legend) blue tones denote positive correlations and red tones, negative correlations. Subscripts in variable labels denote the time point of measurement (B: before, P: peak, A: after).

Table S1. Regression analysis for the in vitro effects of BAFF on virus replication and expansion of EBV-infected LCL B cells after one day of incubation.

<table>
<tr><th>Dependent variable</th><th>Independent variables</th><th>Estimate</th><th>Standard error</th><th>P value</th></tr>
<tr><td rowspan="7">Normalized EBV-PCR value</td><td>(Intercept)</td><td>0.88</td><td>0.06</td><td><0.001</td></tr>
<tr><td>BAFF treatment (y/n)</td><td>0.13</td><td>0.06</td><td>0.041</td></tr>
<tr><td>Immunosuppression (y/n)</td><td>0.19</td><td>0.07</td><td>0.014</td></tr>
<tr><td>Interaction BAFF:Immunosuppression</td><td>-0.16</td><td>0.08</td><td>0.076</td></tr>
<tr><td>Patient effects (Patient 2)</td><td>0.18</td><td rowspan="3">0.06</td><td>0.006</td></tr>
<tr><td>Patient effects (Patient 3)</td><td>0.07</td><td>0.255</td></tr>
<tr><td>Patient effects (Patient 4)</td><td>0.25</td><td><0.001</td></tr>
<tr><td rowspan="7">Normalized LCL B cell count</td><td>(Intercept)</td><td>1.47</td><td>0.14</td><td><0.001</td></tr>
<tr><td>BAFF treatment (y/n)</td><td>0.36</td><td>0.14</td><td>0.017</td></tr>
<tr><td>Immunosuppression (y/n)</td><td>-0.08</td><td>0.16</td><td>0.630</td></tr>
<tr><td>Interaction BAFF:Immunosuppression</td><td>-0.32</td><td>0.20</td><td>0.125</td></tr>
<tr><td>Patient effects (Patient 2)</td><td>-0.52</td><td rowspan="3">0.13</td><td>0.001</td></tr>
<tr><td>Patient effects (Patient 3)</td><td>-0.78</td><td><0.001</td></tr>
<tr><td>Patient effects (Patient 4)</td><td>-0.58</td><td><0.001</td></tr>
</table>
